# An In-Situ Formed Tunneling Layer Enriches the Options of Anode for Efficient and Stable Regular Perovskite Solar Cells

**DOI:** 10.1007/s40820-022-00975-6

**Published:** 2022-12-09

**Authors:** Xuesong Lin, Yanbo Wang, Hongzhen Su, Zhenzhen Qin, Ziyang Zhang, Mengjiong Chen, Min Yang, Yan Zhao, Xiao Liu, Xiangqian Shen, Liyuan Han

**Affiliations:** 1 State Key Laboratory of Metal Matrix Composites, Shanghai Jiao Tong University, Shanghai, 200240 People’s Republic of China; 2 Special Division of Environmental and Energy Science, Komaba Organization for Educational Excellence, College of Arts and Sciences, University of Tokyo, Tokyo, 153-8902 Japan

**Keywords:** Perovskite solar cell, Anode, Halogen migration, In situ tunneling layer

## Abstract

Perovskite solar cells (PSCs) are taking steps to commercialization. However, the halogen-reactive anode with high cost becomes a stumbling block. Here, the halogen migration in PSCs is utilized to in situ generate a uniform tunneling layer between the hole transport materials and anodes, which enriches the options of anodes by breaking the Schottky barrier, enabling the regular PSCs with both high efficiency and stability. Specifically, the regular PSC that uses silver iodide as the tunneling layer and copper as the anode obtains a champion power conversion efficiency of 23.24% (certified 22.74%) with an aperture area of 1.04 cm^2^. The devices are stable, maintaining 98.6% of the initial efficiency after 500 h of operation at the maximum power point with continuous 1 sun illumination. PSCs with different tunneling layers and anodes are fabricated, which confirm the generality of the strategy.

**Supplementary Information:**

The online version contains supplementary material available at 10.1007/s40820-022-00975-6.

## Highlights

- It is first disclosed that the key to the efficient regular Ag-perovskite solar cells (PSCs) is a tunneling layer (silver iodide, AgI) that is in situ formed by the natural reaction between Ag and the migrated iodide.
- Based on the discovery, an ultrathin and uniform tunneling layer can be deposited on the fragile perovskite/charge transport layer to enrich the options of electrodes for efficient and stable regular PSCs.

**Supplementary Information:**

The online version contains supplementary material available at 10.1007/s40820-022-00975-6.

## Graphical abstract

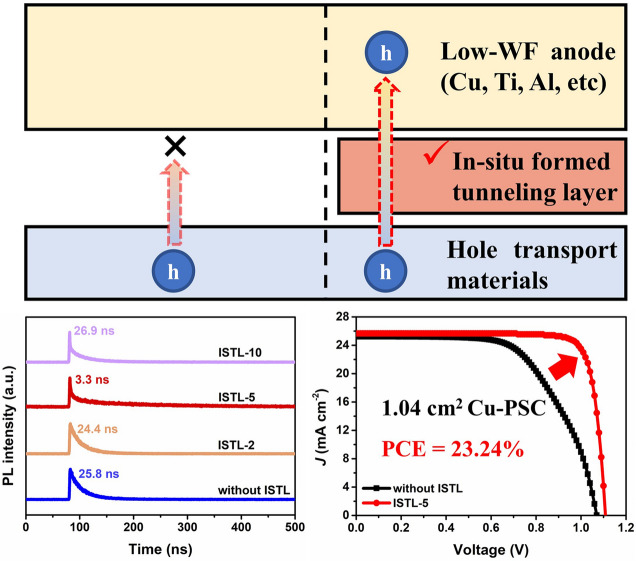

## Introduction

Perovskite solar cells (PSCs), especially for the regular ones, show a sky-rocketing development in power conversion efficiency (PCE) [1–9]. However, the options for the anodes of the regular PSCs are limited due to the requirements of energy level alignment and high conductivity [10–12]. Under this circumstance, the active players are halogen-reactive with high cost, which casts a shadow on the path of regular PSCs toward commercialization [13, 14].

For now, the commonly used anode is gold (Au), which has a high work function (WF) to facilitate the collection of holes from the highest occupied molecular orbital (HOMO) of the hole transport material (HTM) such as 2,2′,7,7′-tetrakis(*N,N*-dip-methoxyphenylamine)-9,9′-spirobifluorene (spiro-OMeTAD) or poly[bis(4-phenyl)(2,4,6-trimethylphenyl)amine] (PTAA) [15]. However, Au was reported to react with the halogen, and the Au atoms could diffuse and form deep-level defects of Au_Pb_ or Au clusters in the perovskite layer, serving as efficient non-radiative recombination centers [16–18]. Moreover, the utilization of Au anode will significantly raise the manufacturing cost of the device [19]. By contrast, the cheap copper (Cu) is an ideal candidate with high resistance to halogen, which has been widely applied to inverted PSCs [20, 21]. Unfortunately, the WF of Cu is too low to efficiently collect the holes from the HTMs [22], and rare work has been published that uses Cu as the anode in regular devices. Interestingly, silver (Ag) shares a similar WF with Cu, but several previous works have obtained PCEs of over 22% using Ag as the anode [23–25]. If we can figure this out, we may enrich the options for the anodes of efficient and stable regular PSCs.

Herein, we disclose that the key to the efficient regular Ag-PSCs is the tunneling layer (silver iodide, AgI) that is in situ formed by the natural reaction between Ag and the migrated iodide, which temporarily eliminates the Schottky barrier at the interface and substantially reduces the non-radiative charge recombination. This finding is important because it had been hard to form an ultrathin and uniform tunneling layer at the interface of HTM and anode without destroying perovskite or HTM [26, 27]. However, the natural reaction will not stop and the efficiency of tunneling will be discounted with the increase in the AgI thickness, which irreversibly undermines the charge collection as well as the operational stability of regular Ag-PSCs [28, 29].

To exploit the unique strength of this natural reaction without side effects, we first deposit an ultrathin layer of Ag atop the HTM, which will be automatically transformed into the tunneling layer under the iodide migration from the perovskite. Then, the anode of Cu is evaporated to complete the fabrication of the device. A champion PCE of 23.24% with an aperture area of 1.04 cm^2^ was achieved for the regular Cu-device using AgI as the tunneling layer. We sent one of the devices for certification at the Shanghai Institute of Microsystem and Information Technology (SIMIT) and obtained an efficiency of 22.51% (average value from a forward scan PCE of 22.28% and a reverse scan PCE of 22.74%). Moreover, the encapsulated devices retain 98.6% of the initial PCEs at the maximum power point (MPP) under continuous 1 sun illumination for 500 h. Other devices using different tunneling layers (cadmium iodide, CdI_2_; tin iodide, SnI_4_) and anodes (titanium, Ti; aluminum, Al) were fabricated and all of them showed decent performances (PCE > 22% under reverse scan), which confirms that our strategy enriches the options for the anodes of regular PSCs.

## Experimental Section

### Materials and Reagents

All the chemicals were brought from companies without further purification. Dispersing SnO_2_, 15% in H_2_O colloidal dispersion, is obtained from Alfa Aesar. Deionized water, dimethylformamide (DMF, 99.8%), dimethyl sulfoxide (DMSO, 99.9%), 4-tert-butylpyridine (tBP, 96%), bis(trifluoromethane)sulfonimide lithium salt (Li-TFSI, 99.95%), PTAA, toluene (99.8%), acetonitrile (ACN, 99.8%), isopropanol (IPA, 95%), acetone, and chlorobenzene (CB, 99.8%) are obtained from Sigma-Aldrich. CH(NH_2_)_2_I (FAI, 98%), CH_3_NH_3_I (MAI, 98%), CH_3_NH_3_Br (MABr, 98%), CH_3_NH_3_Cl (MACl, 98%), CsBr (> 99%), and PbI_2_ (99.99%) are obtained from Tokyo Chemical Industry. Spiro-OMeTAD (99.8%) is obtained from Ningbo Borun New Material Technology Co., LTD. Ag, Au, Ti, Al, Cd, and Sn with a purity of 99.999% are obtained from Beijing Dream Material Technology Co. LTD.

### Fabrication of Perovskite Solar Semi-devices

The semi-device represents the structure of anti-reflection layer/glass/fluorine-doped tin oxide(FTO)/SnO_2_/perovskite/HTM. A precursor solution composed of methyl isobutyl ketone and silicon dioxide nanoparticles was brought from Shanghai Juanrou Newtech Co. LTD and diluted by IPA (1:1/v:v). The diluted solution was spin-coated atop the glass side of FTO substrates at 3000 rpm for 30 s, followed by the thermal annealing process at 120 °C for 2 h to remove the solvent and obtain the anti-reflection layer. The FTO were etched with zinc powder and 6 M aqueous hydrochloric acid for 15 s for patterned substrates and then sonicated with detergent, deionized water, ethanol, acetone and IPA for 15 min, respectively. The FTO substrates were cleaned with ultraviolet ozone under dry air for 20 min. Then, SnO_2_ dispersion (1:8.5/v:v in deionized water) was spin-coated atop substrates at 3000 rpm for 30 s and annealed under ambient atmosphere at 150 °C for 30 min to form the electron transport material (ETM). After treated by ultraviolet ozone under dry air for 20 min, the samples were transferred to the nitrogen glove box immediately. For (FA,MA)PbI_3_ perovskite fabrication, 1.5 M PbI_2_ precursor in DMF:DMSO (9:1/v:v) was spin-coated on SnO_2_ ETM at 1500 rpm for 40 s and annealed at 70 °C for 1 min. A solution of FAI:MAI:MACl (90 mg:12 mg:9 mg in 1 mL IPA) was spin-coated on PbI_2_ at 1800 rpm for 30 s. For (FA,MA)Pb(I,Br)_3_ perovskite fabrication, 1.5 M PbI_2_ precursor in DMF:DMSO (9:1/v:v) was spin-coated on SnO_2_ ETM at 1500 rpm for 40 s and annealed at 70 °C for 1 min. A solution of FAI:MABr:MACl (90 mg:9 mg:9 mg in 1 mL IPA) was spin-coated on PbI_2_ at 1500 rpm for 30 s. The films were annealed at 150 °C for 15 min under ambient air (30–34% relative humidity, RH). The annealed films were transferred to the nitrogen glove box and cooled down. For the Cs-based perovskite, the perovskite precursor was prepared by dissolving 219.3 mg FAI, 619.5 mg PbI_2,_ and 0.05 mg CsBr stoichiometrically into 1 mL DMF/DMSO (4:1/v:v). The perovskite precursor was spin-coated onto SnO_2_ ETM at 1000 rpm for 10 s and 6000 rpm for 50 s. 150 μL CB was dripped onto the center of the film at 15 s before the end of the spin-coating process. The films were annealed at 150 °C for 20 min in the glove box. 72.3 mg spiro-OMeTAD or 10 mg PTAA HTM with additives of 17.5 μL Li-TFSI (520 mg Li-TFSI in 1 mL ACN) and 28.8 μL tBP in 1 mL CB or toluene was spin-coated atop perovskite film at 3000 rpm for 30 s.

### Fabrication of In-situ Formed Tunneling Layer (ISTL) and Anode

For PSCs based on Ag and Au anodes, the sample was transferred to dark and dry air box for fully oxidation of HTM for 24 h. Then, 80 nm Ag and Au were thermally evaporated as anodes using a shadow mask with a substrate cooling system. For PSCs with an ISTL, an ultrathin Ag, Cd, or Sn layer was thermally deposited on a HTM in rotation and with a vacuum degree of < 2 × 10^−7^ Torr, deposition rate of *ca.* 0.05 Å per seconds, and substrate temperature of < 20 °C. Then, the device was transferred to dry air box, which not only oxidized the HTM but also allowed the halogen components to migrated and reacted with the Ag, Cd, or Sn to form an ISTL. The 80 nm Cu or Al was thermally evaporated as anodes, and high-melting-point Ti was prepared by electron beam deposition to prevent the heat-induced damage [30]. The 0.09 and 1.04 cm^2^ masks were used to define the active areas during measurements.

### Characterization of Materials and Device Performances

**Fig. 1:**
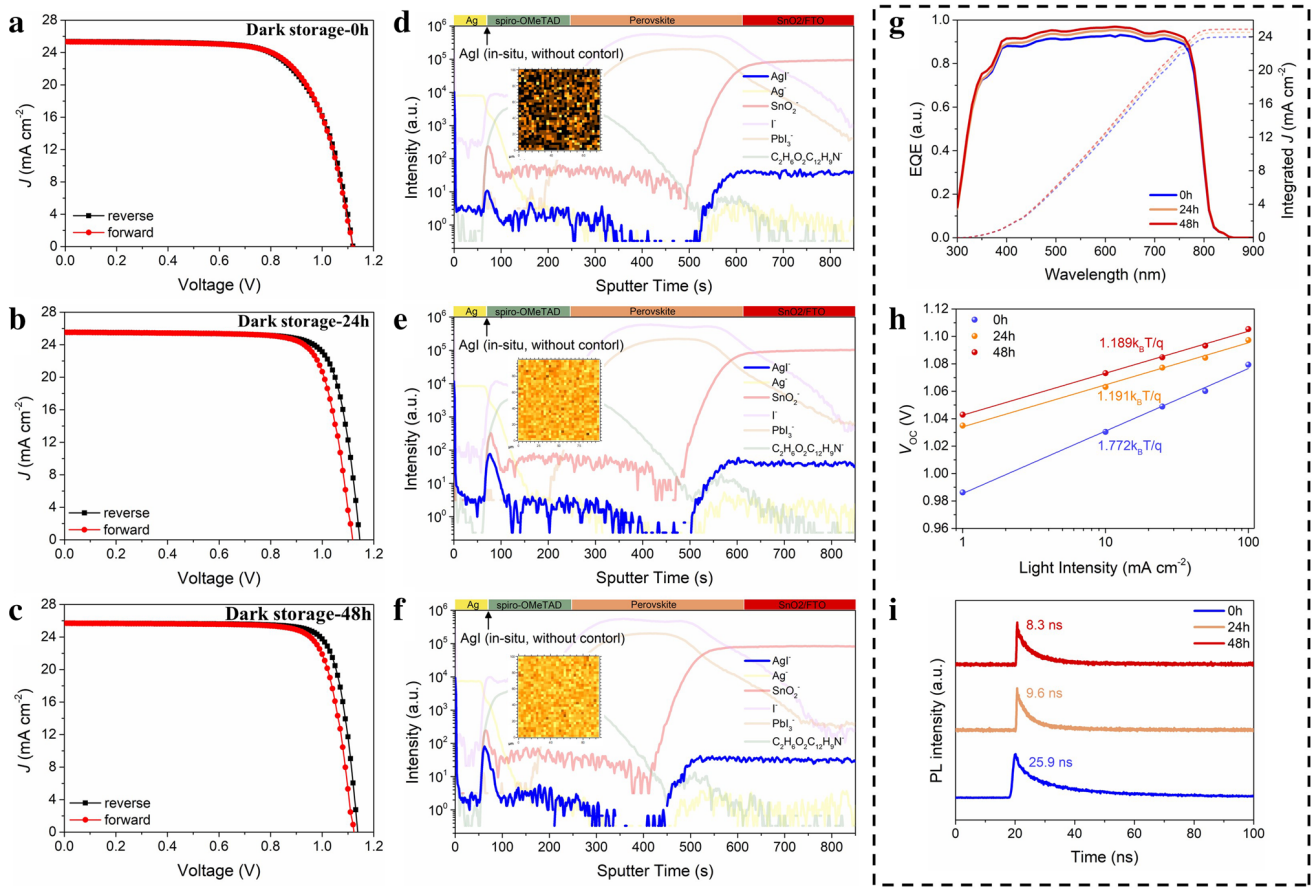
*In situ* formation of AgI tunneling layer and its effects on device performances. **a**–**c** *J–V* curves of the champion regular Ag-PSC and **d**–**f** the depth profiles of ToF–SIMS (Inset: 2D elemental mapping of AgI^−.^ signal at Ag/spiro-OMeTAD interface) after being stored for 0, 24 and 48 h. **g** The IPCE and **h** ideality factor of the corresponding devices. **i** The evolution of TRPL for glass/SnO_2_/(FA,MA)PbI_3_ perovskite/spiro-OMeTAD/Ag sample. Notes all devices in the figure were stored under dark and dry environment (< 10% RH)

The current density–voltage (*J–V*) curves of PSCs were measured under a simulated solar source and AM 1.5G (100 mW cm^−2^, WXS-155S-10, Wacom Denso Co., Japan) with a Keithley 2400 digital source meter. The simulated solar source was calibrated by a standard silicon reference cell, certified by the calibration, standards, and measurement team at the research center for photovoltaics in AIST, Japan, with a spectral mismatch of < 3%. The *J–V* measurements were carried out from 1.2 to − 0.2 V (reverse scan) and − 0.2 to 1.2 V (forward scan). The reason for testing Cu-PSCs after 48 h of storage is to ensure the consistency with the testing conditions of Ag-PSCs in Fig. 1a–c. The corresponding incident photo-to-electron conversion efficiency (IPCE) spectra were measured on a monochromatic incident light with 1 $\times$ 10^16^ photon cm^−2^ and the alternating current mode. By utilizing filters to regulate the light intensity, the ideality factor results were measured on the simulated solar source. The time-of-flight secondary ion mass spectroscopy (ToF–SIMS) measurements were based on ION TOF SIMS 5–100 (Germany) by utilizing a stable Cs^+^ ion beam as the ion beam to peel off devices with an area of 100 × 100 μm^2^. The time-resolved photoluminescence (TRPL) results were measured on a FLS1000 system (Edinburgh, English) and fitted by the bi-exponential formula (Eqs. 1 and 2) [31]. The X-ray diffraction (XRD) patterns were characterized by a multifunctional x-ray diffractometer (D8 Advance Da Vinci, Germany) from 10 to 60 degree with a scan speed of 0.3 degree per second. The atomic force microscopy (AFM) measurements were carried out on a FastScan Bio (USA). The ultraviolet photoelectron spectroscopy measurement (UPS) and X-ray photoelectron spectroscopy (XPS) were measured with an AXIS UltraDLD by Al K $\mathrm{\alpha }$ X-ray source (China). The signals were normalized to deduct the interference caused by the total internal reflection of ISTL/anode interface [32]. A field emission scanning electron microscopy (FESEM, JEOL JEM-7800 Prime, China) was employed to investigate the structure and element distribution of samples. The images of transmission electron microscopy (TEM) and magnified high-resolution scanning TEM (HR-STEM) were obtained by a Talos F200X G2 (USA) with an energy-dispersive x-ray spectroscopy (EDS) equipment, and the sample was prepared by the focused ion beam (FIB) technique (GAIA3, Czech Republic). To exclude the effect of the environment and the damage by the high-energy FIB, an electron beam-deposited platinum (Pt) and ion beam-deposited Pt were coated (GAIA3, Czech Republic). Moreover, a 2-μm carbon (C) layer was further coated atop Pt layers. To reduce the negative effect of heat on sample preparation, a small voltage of FIB (5 kV) was chosen. The electrochemical impedance spectroscopy was conducted on an electrochemical workstation (Zahner, Germany) in dark condition with 0.9 V bias around 1 Hz to 1 MHz and the equivalent circuit employed to fit the spectra.

1 $$f\left( x \right) = A_{1} \exp \left( {\frac{ - t}{{\tau_{1} }}} \right) + A_{2} \exp \left( {\frac{ - t}{{\tau_{2} }}} \right) + B$$

2 $$\tau_{ave} = \left( {\frac{{A_{1} }}{{A_{1} + A_{2} }}\tau_{1}^{2} + \frac{{A_{2} }}{{A_{1} + A_{2} }}\tau_{2}^{2} } \right)/\left( {\frac{{A_{1} }}{{A_{1} + A_{2} }}\tau_{1} + \frac{{A_{2} }}{{A_{1} + A_{2} }}\tau_{2} } \right)$$

### Operational Stability Test

For the stability characterization, the spiro-OMeTAD was replaced by PTAA to achieve better stability. The stability of the encapsulated PSCs was carried out on a solar cell light resistance testing system and measured at the MPP under 1-sun illumination (100 mW cm^−2^). The PCEs of above PSCs were measured every 12 h under reverse *J–V* scan.

### Fitting Method of the Average Diffusion Time (***t***_ave_) of Iodine Ions through the HTM with Different Thicknesses

To determine the *t*_ave_ of iodine ions, the diffusion of iodine ions is fitted by the model of attenuating film source based on the one-dimensional diffusion equation. The procedure can be described as follows. First, as the number of iodine ions is limited, the perovskite films can be considered as an attenuating film source of iodine ions, and the HTM can be considered as a diffusion medium of iodine ions. We used the 2nd Fick’s law to get the concentration distribution ($\rho$) of diffusing iodine ions in the regions of perovskite and HTM based on Eq. 3.

3 $$\frac{\partial C}{{\partial t}} = D\frac{{\partial^{2} C}}{{\partial x^{2} }}$$

where *C* is the concentration of iodine ions in perovskite; *D* is the diffusion coefficient of iodine ions in the HTM region, normalized by the initial concentration (*t* = 0) in perovskite. *t* = 0 is determined by the first contact of perovskite and HTM.

We then define the initial conditions by considering that, at *t* = 0, there is no iodine ions in the HTM ($x\ne 0$, *C* = 0). In the meantime, *C* equals to 1 in the whole perovskite region ($x=0$). To get the boundary conditions, we assumed that there was no diffusion in or out of the perovskite at the perovskite/FTO interface and the diffusion coefficient is independent of the concentration of iodine ions. The numerical solution of Gaussian distribution is described as Eq. 4.

4 $$\rho \left( {x, t} \right) = \frac{M}{{4\sqrt {\pi Dt} }}\exp \left( { - \frac{{x^{2} }}{4Dt}} \right)$$

where *M* is the mass of iodine ions per unit area, and the average diffusion length of iodine ions toward one dimension is marked as *d*, which is consistent with the thickness of the HTM. The relation between d and *t*_ave_ is shown in Eq. 5.

5 $$d = 2\sqrt {Dt_{{{\text{ave}}}} } \;\text{or}\;t_{{{\text{ave}}}} = \frac{{d^{2} }}{4D}$$

According to previous works [33–35], PSCs system based on the device structure of SnO_2_/FAPbI_3_/spiro-OMeTAD had been evaluated, which is similar with this work and the *D* was calculated to be *ca.* 2.23 $\times$ 10^–13^ cm^2^ s^−1^. To verify the effect of the thickness of HTM on the diffusion, the range of *d* is between 100 and 300 nm. The range is selected considering that the HTM is required to fully cover the perovskite without increasing the series resistance (*R*_s_) of the devices.

### Evaluation of the Formation Energy of Metal Halide

The formation energy of metal halide was evaluated by the standard metal atom-halogen atom binding energy of 1 mol metal atoms and 1 mol halogen atoms. For example, the formation energy of AgI was equal to the difference of energy that Ag metal with 1 mol Ag atoms and I element with 1 mol I atoms decomposed into independent 1 mol Ag and I atoms and energy AgI with 1 mol Ag and I atoms decomposed into independent 1 mol Ag and I atoms. In other words, the formation energy of AgI is equal to $E_{{{\text{Ag}} - {\text{I}}}} - E_{{{\text{Ag}} - {\text{Ag}}}}$ of -71 kJ mol^−1^, as shown in Eq. 6. A similar method is utilized in Eqs. 7 and 8 to evaluate the formation energy of CdI_2_ and SnI_4_.

## Results and Discussion

### Mechanism of Efficient Regular Ag-PSCs

We fabricated simplified devices with the structure of glass/ FTO/SnO_2_/(FA,MA)PbI_3_(FA as formamidinium, MA as methylammonium)/spiro-OMeTAD/Ag and no passivation layers were used. Before the deposition of Ag, the semi-devices were stored in the dry box for 24 h to realize the oxidization of spiro-OMeTAD. The environment conditions of storage are under < 3% RH, 20 °C and in a dry box if not specified. Figure 1a-c shows the evolution of the *J–V* curves of the champion regular Ag-PSC among twenty cells during 48 h of storage in the dark. The detailed photovoltaic parameters are summarized in Table S1. Obviously, the PCE of the device presents a spontaneous improvement from 19.12% to 23.86% for the reverse scan (19.50% to 22.87% for the forward scan), which is mainly contributed to the enhancement of the fill factor (FF). To further illustrate the evolution on charge transport, the corresponding IPCE spectra and ideality factor were investigated. A slight increase in the integrated *J* is observed, which is consistent with that in the short-circuit *J* (*J*_SC_) (Fig. 1g and Table S1). Meanwhile, the ideality factor is decreased from 1.772 to 1.189, indicating the suppression of the non-radiative recombination loss (Fig. 1h) [36, 37]. The statistics of the twenty cells show a significant improvement in the average PCE by 25% with that of FF by 23% (Fig. S1), and we also observed similar phenomenon in the PSCs with another record-maker perovskite, (FA,MA)Pb(I,Br)_3_, indicating its universality in regular Ag-PSCs (Fig. S3 and Table S2). From the perspective of the equivalent circuit of a solar cell with parasitic resistance (details shown in Note S1 and Fig. S2), the large increase in FF without obvious changes in *J*_SC_ and *V*_OC_ can be explained by the significantly reduced *R*_s_ with a quasi-constant shunt resistance (*R*_sh_) [28, 38, 39].

To unravel the working mechanism in the PCE enhancement, we performed the ToF–SIMS on the regular Ag-PSCs at different stages (0, 24, and 48 h). It was found that the signal of AgI^−^ (blue line) at the interface of spiro-OMeTAD/Ag for the device after 24 h of storage is nearly ten times the intensity of the fresh one, as shown in Fig. 1d–f. In the meantime, the spatial distribution of other elements remains unchanged. The corresponding ToF–SIMS 2D elemental mappings at this interface further confirmed the formation of a fully-covered AgI layer after 24 h of storage and the signals are slightly enhanced after 48 h (Insets, Fig. 1d–f). The above results indicate that the halogen-migration-induced AgI with the wide band gap is perhaps the reason for the PCE improvement. To exclude other possibilities, the influence of perovskite and other interfaces on the charge transport with varying storage times was systematically investigated. As shown in Fig. S4, the crystal quality of the perovskites characterized by the XRD shows no difference after 72 h of storage. Moreover, the evolution of TRPL spectra of glass/SnO_2_/(FA,MA)PbI_3_ perovskite/spiro-OMeTAD sample is shown in Fig. S5; the carrier lifetimes remain at *c.a.* 25 ns during 72 h of storage, indicating its negligible contribution to the improvement in charge collection. On the contrary, the charge collection is accelerated by threefold for glass/SnO_2_/(FA,MA)PbI_3_ perovskite/spiro-OMeTAD/Ag sample after 48 h of storage (Fig. 1i).

**Fig. 2:**
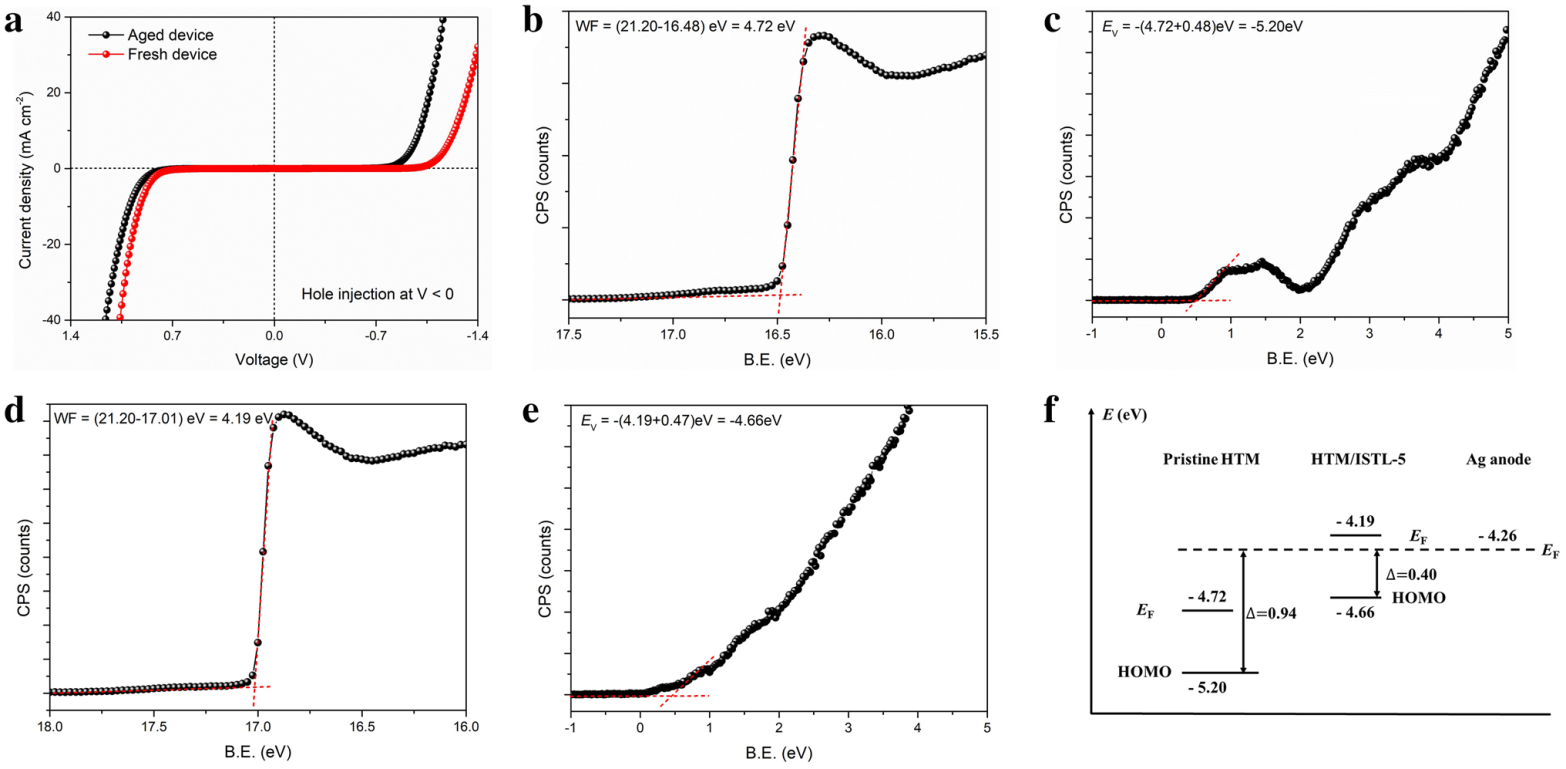
Elimination of the Schottky barrier at HTM/Ag interface by naturally formed AgI tunneling layer. **a** The *J–V* characteristics under dark for the fresh Ag-PSCs and the aged Ag-PSCs after 24-h of storage. **b, d** The high binding energy cutoff region and **c, e** low binding-energy region of UPS results of spiro-OMeTAD **b, c** without or **d, e** with AgI. **f** The schematic of the change in the energy level. The aged Ag-PSCs in **2a** and samples in **2b-e** were stored under dark and dry environment (< 10% RH)

We further investigated the ability of AgI to inject holes from the HTM into Ag anode. A comparison of the *J–V* characteristics between 24-h-stored Ag-PSCs and fresh Ag-PSCs is shown in Fig. 2a. The aged device presents a symmetric *J–V* characteristics, which represents the formation of ohmic contact between the HTM and Ag anode with AgI. To directly show the change in the energy level originating from the AgI, we evaporated 5-nm Ag atop the HTM and performed UPS after 24 h of storage (Fig. 2b–e) and the schematic of the change is shown in Fig. 2f. Compared with the pristine spiro-OMeTAD, the energy barrier between the HOMO of the HTM and the WF of the Ag anode is greatly reduced from 0.94 eV to 0.40 eV, which guarantees the effective tunneling process. The above results are consistent with the performance evolution of the devices in Fig. 1. Therefore, we could conclude that the efficient regular Ag-PSCs are realized by the naturally formed AgI tunneling layer between the HTM and anode.

Unfortunately, the reaction between halogen and Ag will not stop and we observed that the signal of Ag 3*d* core level shifts to higher bind energy and the intensity of I 3*d* core level continuously increases as the storage time prolongs (Fig. S6). The energy-favored reaction of Ag and halogen (Eq. 6, detail seen in Experimental Section) are uncontrollable and will surely compromise the long-term stability of PSCs, as was reported by previous works [34, 40, 41]. The operational stability of the encapsulated regular Ag-PSCs was measured at the MPP with continuous 1-sun illumination and only 15.1% of the initial PCE retained after 360 h. Even if the anode is replaced by Au, the PCE dropped by 70.7% after 500 h of operation (Fig. S7). The *J–V* curves of champion Au-PSC are shown in Fig. S8.

6 $${\text{Ag}}^{0} + {\text{I}}^{0} \to {\text{AgI }}E_{{{\text{Ag}} - {\text{I}}}} - E_{{{\text{Ag}} - {\text{Ag}}}} = - 71{\text{ kJ mol}}^{ - 1}$$

### Controllable Formation of the Tunneling Layer and Its Effects

**Fig. 3:**
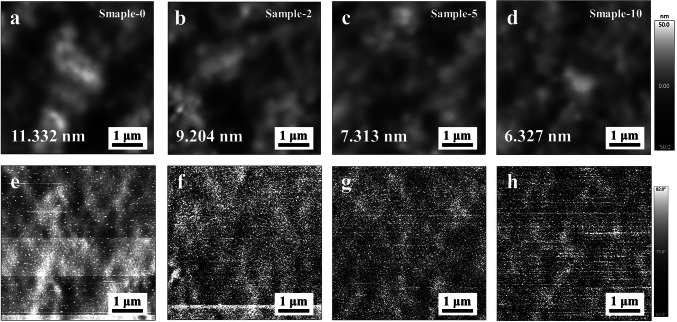
Characterization of the ISTL (AgI) on the surface of spiro-OMeTAD. **a-d** The xy-plane film morphology of spiro-OMeTAD and spiro-OMeTAD/AgI fabricated with different thickness of Ag (2, 5 and 10 nm), respectively, measured by atomic force microscopy (AFM). **e–h** The corresponding phase images. All the samples were stored in the dark for 24 h before measurement

To utilize the ISTL without further corrosion, an ultrathin layer (0 to 10 nm) of Ag was deposited atop spiro-OMeTAD before the evaporation of anode and the samples were denoted as sample-0, sample-2, sample-5, and sample-10, respectively. Compared with sample-0 (Fig. 3a), the surface roughness of the other three fresh samples gradually decreases from 11.332 nm to 9.582, 9.291, and 8.838 nm, indicating that the evaporated Ag is down to the valleys of the HTMs (Fig. S9). To convert the Ag to AgI, the samples were stored for 24 h because the performances of the aged Ag-PSCs for 24 and 48 h are close (Fig. 1b, c). In addition, we estimated the *t*_ave_ of iodine ions by Fick’ law to investigate the effect of the thickness of spiro-OMeTAD HTM on the diffusion of iodine ions, as summarized in Table S3 (calculation details shown in Experimental Section). The values of *t*_ave_ are all less than one-third of an hour as the thickness of spiro-OMeTAD HTM ranges from 100 to 300 nm. This result indicated that a 24-h storage is sufficient for halogen migration to form the ISTL under general conditions. After the storage, we performed the XPS for the four samples and found that the characteristic peaks of Ag 3*d* core level for sample-2, sample-5 and sample-10 shifted from 368.29 and 374.29 eV (Fig. S6, blue line) to 368.17 and 374.17 eV (Fig. S10), respectively, indicating the conversion from Ag to AgI, which is consistent with the results of ToF–SIMS (Fig. 1e) [28, 42]. Moreover, the surface roughness further decreases to 9.204, 7.313, and 6.327 nm, respectively (Fig. 3b–d). The “smooth effect” can be explained by the higher undercooling and heterogeneous nucleation rates in valleys by thermal evaporation and possible halogen migration through the surface to interconnect the ultrathin Ag layer [43–45]. The corresponding phase images confirmed the clear contrast of mechanical properties after the formation of AgI (Fig. 3e–h).

**Fig. 4:**
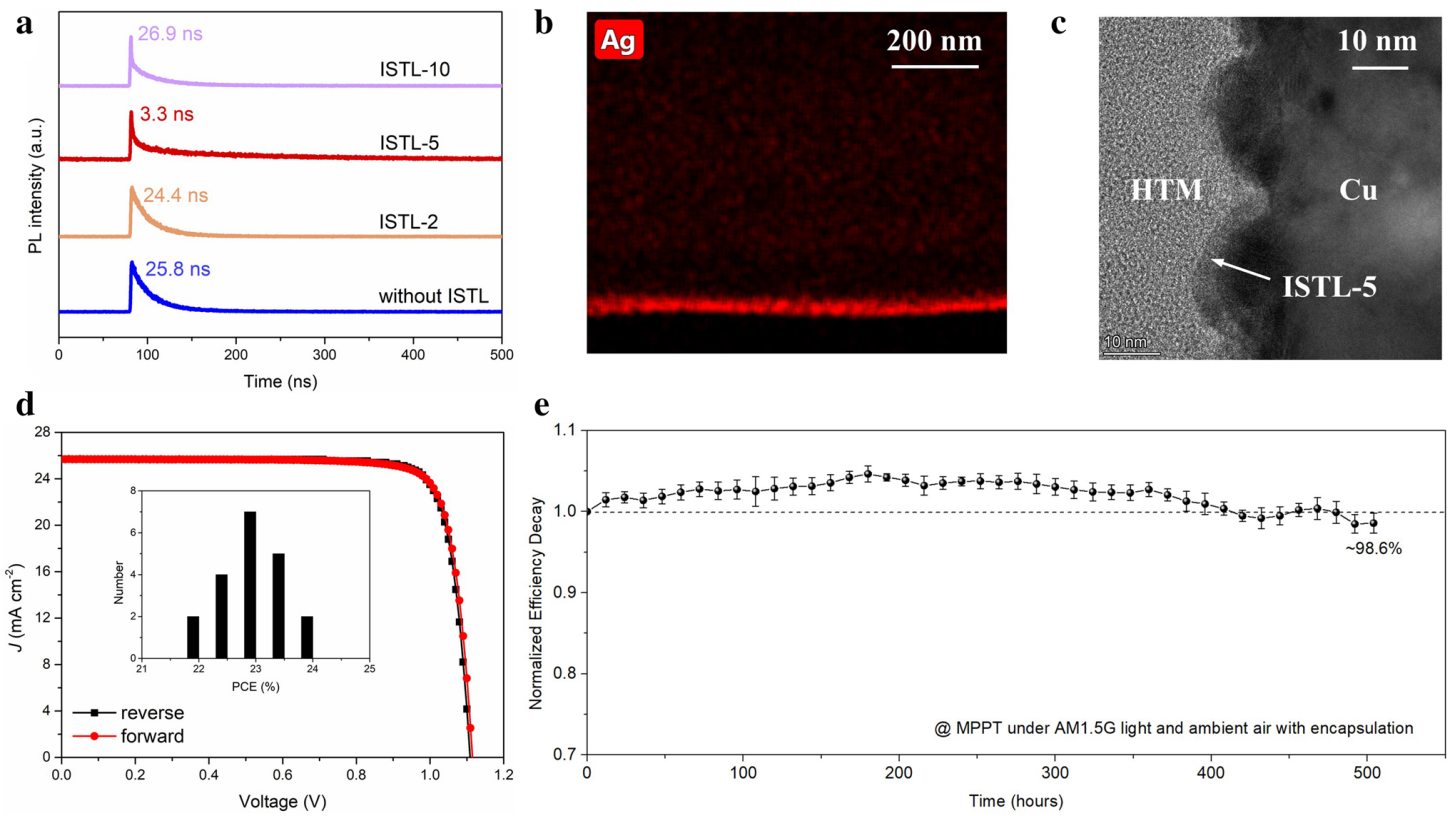
Performances of the devices with ISTL (AgI) and Cu anode. **a** The TRPL results of glass/perovskite/spiro-OMeTAD/ISTLs/Cu. **b** The TEM image (inset: HR-STEM image of Cu/ISTL-5/HTM structure) and **c** corresponding EDS mapping of Ag element. **d** The *J–V* curves of the champion device with ISTL-5 (Inset: the histogram of average PCE value of twenty devices with ISTL-5). **e** The operational stability of the encapsulated devices with ISTL-5. The scale bar is 200 nm. The TEM and HR-STEM sample was prepared by FIB technique and the Pt and C was acted as protective multilayer to exclude the effect of the environment. The initial PCEs are 22.0 ± 0.2%, and the error bars denote standard deviations for five individual devices

To examine the effect of ISTL in the samples that use anodes with inappropriate WF, we performed TRPL on glass/perovskite/spiro-OMeTAD/ISTLs/Cu (Fig. 4a). It was found that when the ISTL is formed by 5 nm Ag (ISTL-5), the average lifetime for carriers suddenly drops from 25.8 to 3.3 ns, indicating an effective tunneling of the carriers. We also noticed that when the ISTL is too thin (ISTL-2) or too thick (ISTL-10), the carrier collection is obviously affected by poor coverage or high resistance. The proposition was further confirmed by the optical charge recombination resistance (*R*_rec_) of the Cu-PSC with ISTL-5, as shown in Fig. S11. The uniform formation of an ISTL-5 is further observed by the TEM with EDS and the SEM measurements (Figs. 4b, c and S12–S13). To make it clearer, the magnified HR-STEM was given, and the thickness of ISTL-5 is *ca.* 5.5 nm (Fig. 4b, inset). Accordingly, we fabricated the full devices using Cu as the anodes with different thickness of ISTLs and the performances are detailed in Table S4, which is consistent with the results of TRPL. The hysteresis-free *J–V* curves of the champion device with ISTL-5 are shown in Fig. 4d, and the inset is the histogram of average PCE value of twenty devices with ISTL-5. A PCE of 23.64% was obtained with an open-circuit voltage (*V*_OC_) of 1.11 V, *J*_SC_ of 25.68 mA cm^−2^, and FF of 82.95% under reverse scan, and these performances were basically consistent in the first 48 h (Table S5). On the contrary, the devices without ISTL or with ISTL-2, ISTL-10 exhibited much poorer performances due to the insufficient carrier collection and severe charge recombination (Fig. S14 and Table S4). We also tried to directly evaporate the AgI atop the HTM to fabricate the devices (AgI-PSCs), and the champion AgI-PSC shows a PCE of 22.2% (Fig. S15). The larger *R*_s_ of 3.9 Ohm cm^2^ and lower FF of 80.4% are probably due to the thermal dissociation and then the lower uniformity of AgI by high-temperature ex situ deposition method [46, 47]. Due to the much higher halogen resistance of Cu than Ag, the encapsulated devices with ISTL-5 retain 98.6% of the initial efficiency after 500 h operating at MPP under 1-sun illumination (Fig. 4e).

To investigate the scalability of this ISTL strategy, we fabricated the devices using ISTL-5 and Cu anode with an aperture area of 1.04 cm^2^, and a champion PCE of 23.24% was achieved under reverse scan, with a *V*_OC_ of 1.13 V, *J*_SC_ of 25.73 mA cm^−2^ and FF of 79.93% (Fig. S16). We sent one of the devices to an independent certified laboratory (SIMIT); a certified efficiency of 22.74% is obtained under reverse scan (Fig. S17). As far as we know, this is the highest value for the PSCs using non-noble metals as the anode (cell area > 1 cm^2^).

### Generality of ISTL Strategy

**Fig. 5:**
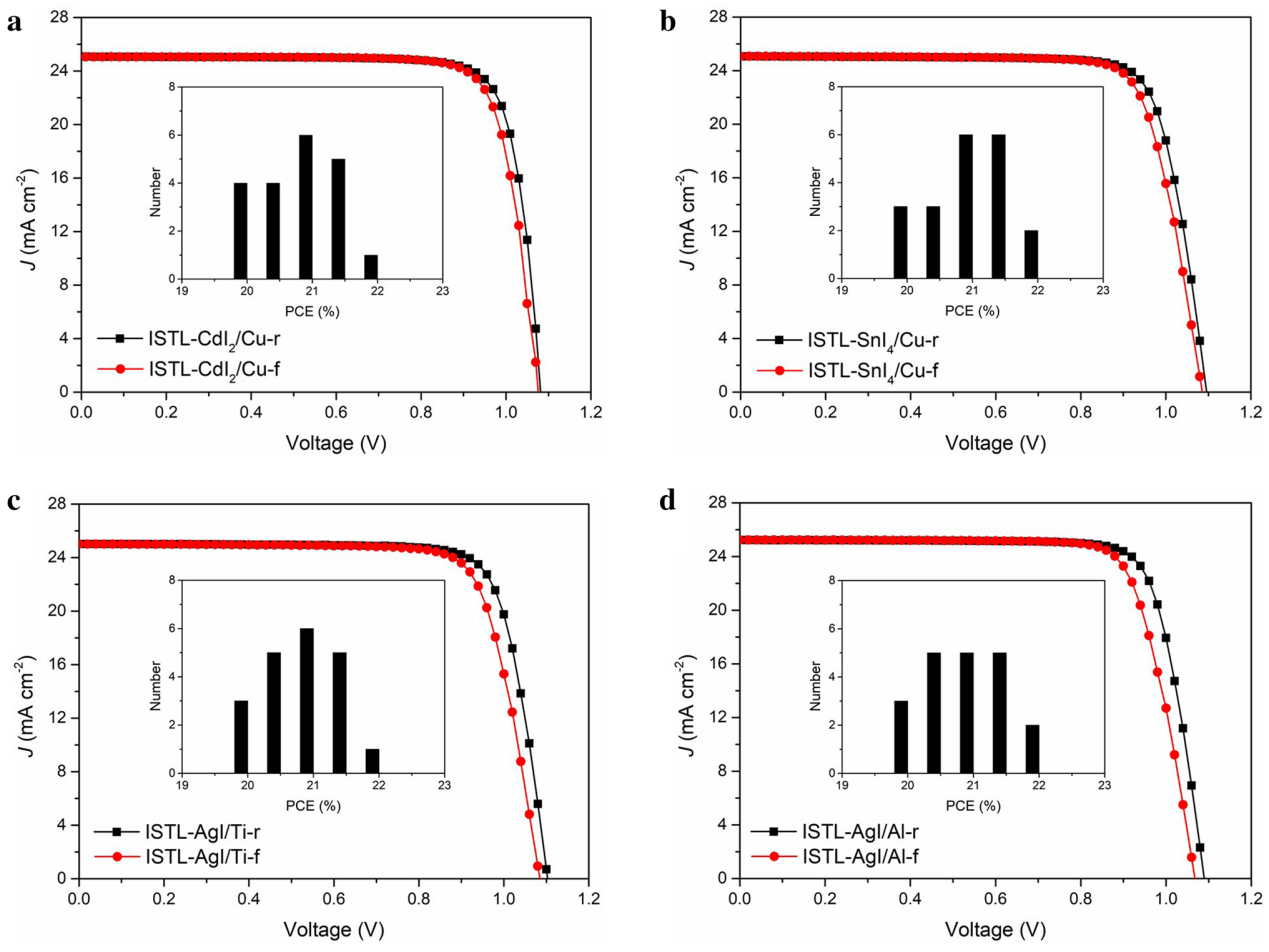
*J–V* curves of champion PSCs with different ISTLs and low-WF anodes under reverse and forward scan. **a** ISTL-CdI_2_/Cu, **b** ISTL-SnI_4_/Cu, **c** ISTL-AgI/Ti and **d** ISTL-AgI/Al anode. Notes that the thickness of ISTLs and anodes is fixed at 5 nm and 80 nm, respectively. The insets in **a–d** are the histograms of average PCE value of twenty 0.09 cm^2^-devices with different ISTLs and anodes

To illustrate the generality of the strategy, the interface reaction layer of Ag was replaced by halogen-active Cd (Eq. 7) and Sn (Eq. 8) with the thickness of 5 nm (detail seen in Experimental Section), and the Cu anode was replaced by Ti (WF = 4.33 eV) and Al (WF = 4.28 eV) [48, 49]. All types of devices show a champion PCE of over 22% under reverse scan (Fig. 5 and Table S6). Moreover, the monohalide perovskite (FA,MA)PbI_3_ was replaced with other perovskite, including mixed halide perovskite (FA,MA)Pb(I,Br)_3_ and Cs-based perovskite FA_0.85_Cs_0.15_Pb(I_0.95_Br_0.15_)_3_. All PSCs show a champion PCE of over 23% under reverse scan (Fig. S18).

7 $${\text{Cd}}^0 + {\text{I}}^0 \to {\text{CdI}}_{2} \quad E_{{{\text{Cd}} - {\text{I}}}} - E_{{{\text{Cd}} - {\text{Cd}}}} = - 131\,{\text{kJ mol}}^{ - 1}$$

8 $${\text{Sn}}^0 + {\text{I}}^0 \to {\text{SnI}}_{4} \quad E_{{{\text{Sn}} - {\text{I}}}} - E_{{{\text{Sn}} - {\text{Sn}}}} = - 46.9\,{\text{kJ mol}}^{ - 1}$$

## Conclusions

IN summary, we disclosed the mechanism behind efficient regular Ag-devices. Based on this discovery, we make it possible and easy to fabricate an ultrathin but uniform tunneling layer atop the HTM to enrich the options for the anodes of regular PSCs. Finally, efficient and stable regular PSCs are obtained using AgI as the tunneling layer and Cu as the anode, which achieved a certified highest efficiency in PSCs that uses non-noble metal anode (cell area > 1 cm^2^). Our strategy also shows extensive opportunities by designing the halogen-reactive layers and the anodes, which offers a promising future to further improve the stability and lower the cost without sacrificing the efficiency.

### Supplementary Information

Below is the link to the electronic supplementary material.

Supplementary file1 (PDF 2238 KB)

## References

[1] Jiang Q, Zhao Y, Zhang X, Yang X, Chen Y (2019). Surface passivation of perovskite film for efficient solar cells. Nat. Photon..

[2] Hui W, Chao LF, Lu H, Xia F, Wei Q (2021). Stabilizing black-phase formamidinium perovskite formation at room temperature and high humidity. Science.

[3] Jeong J, Kim M, Seo J, Lu HZ, Ahlawat P (2021). Pseudo-halide anion engineering for alpha-FAPbI_3_ perovskite solar cells. Nature.

[4] Min H, Lee D, Kim J, Kim G, Lee KS (2021). Perovskite solar cells with atomically coherent interlayers on SnO_2_ electrodes. Nature.

[5] Yoo JJ, Seo G, Chua MR, Park TG, Lu YL (2021). Efficient perovskite solar cells via improved carrier management. Nature.

[6] Peng J, Kremer F, Walter D, Wu Y, Ji Y (2022). Centimetre-scale perovskite solar cells with fill factors of more than 86 per cent. Nature.

[7] Li NX, Niu XX, Li L, Wang H, Huang ZJ (2021). Liquid medium annealing for fabricating durable perovskite solar cells with improved reproducibility. Science.

[8] Wang YB, Wu TH, Barbaud J, Kong WY, Cui DY (2019). Stabilizing heterostructures of soft perovskite semiconductors. Science.

[9] Su H, Lin X, Wang Y, Liu X, Qin Z (2022). Stable perovskite solar cells with 23.12% efficiency and area over 1 cm^2^ by an all-in-one strategy. Sci. China Chem..

[10] Xiao JW, Shi CB, Zhou CX, Zhang DL, Li YJ (2017). Contact engineering: electrode materials for highly efficient and stable perovskite solar cells. Sol. RRL.

[11] Wang SH, Sakurai T, Wen WJ, Qi YB (2018). Energy level alignment at interfaces in metal halide perovskite solar cells. Adv. Mater. Interfaces.

[12] Xu YM, Lin ZH, Wei W, Hao Y, Liu SZ (2022). Recent progress of electrode materials for flexible perovskite solar cells. Nano-Micro Lett..

[13] Boyd CC, Cheacharoen R, Leijtens T, McGehee MD (2019). Understanding degradation mechanisms and improving stability of perovskite photovoltaics. Chem. Rev..

[14] Li JW, Dong QS, Li N, Wang LD (2017). Direct evidence of ion diffusion for the silver-electrode-induced thermal degradation of inverted perovskite solar cells. Adv. Energy Mater..

[15] Calio L, Kazim S, Gratzel M, Ahmad S (2016). Hole-transport materials for perovskite solar cells. Angew. Chem. Int. Ed..

[16] Cacovich S, Cina L, Matteocci F, Divitini G, Midgley PA (2017). Gold and iodine diffusion in large area perovskite solar cells under illumination. Nanoscale.

[17] Domanski K, Correa-Baena JP, Mine N, Nazeeruddin MK, Abate A (2016). Not all that glitters is gold: metal-migration-induced degradation in perovskite solar cells. ACS Nano.

[18] Shlenskaya NN, Belich NA, Gratzel M, Goodilin EA, Tarasov AB (2018). Light-induced reactivity of gold and hybrid perovskite as a new possible degradation mechanism in perovskite solar cells. J. Mater. Chem. A.

[19] Fagiolari L, Bella F (2019). Carbon-based materials for stable, cheaper and large-scale processable perovskite solar cells. Energy Environ. Sci..

[20] Zhao JJ, Zheng XP, Deng YH, Li T, Shao YC (2016). Is Cu a stable electrode material in hybrid perovskite solar cells for a 30-year lifetime?. Energy Environ. Sci..

[21] Li ZQ, Zhao YZ, Wang X, Sun YC, Zhao ZG (2018). Cost analysis of perovskite tandem photovoltaics. Joule.

[22] Lin X, Cui D, Luo X, Zhang C, Han Q (2020). Efficiency progress of inverted perovskite solar cells. Energy Environ. Sci..

[23] Chiang CH, Kan CW, Wu CG (2021). Synergistic engineering of conduction band, conductivity, and interface of bilayered electron transport layers with scalable TiO_2_ and SnO_2_ nanoparticles for high-efficiency stable perovskite solar cells. ACS Appl. Mater. Interfaces.

[24] He MS, Liang JH, Zhang ZF, Qiu YK, Deng ZH (2020). Compositional optimization of a 2D–3D heterojunction interface for 22.6% efficient and stable planar perovskite solar cells. J. Mater. Chem. A.

[25] Zhang ZF, Wang JL, Lang LZ, Dong Y, Liang JH (2022). Size-tunable MoS2 nanosheets for controlling the crystal morphology and residual stress in sequentially deposited perovskite solar cells with over 22.5% efficiency. J. Mater. Chem. A.

[26] Peng J, Walter D, Ren Y, Tebyetekerwa M, Wu Y (2021). Nanoscale localized contacts for high fill factors in polymer-passivated perovskite solar cells. Science.

[27] Giuliano G, Cataldo S, Scopelliti M, Principato F, Martino DC (2019). Nonprecious copper-based transparent top electrode via seed layer–assisted thermal evaporation for high-performance semitransparent n-i-p perovskite solar cells. Adv. Mater. Technol..

[28] Kato Y, Ono LK, Lee MV, Wang S, Raga SR (2015). Silver iodide formation in methyl ammonium lead iodide perovskite solar cells with silver top electrodes. Adv. Mater. Interfaces.

[29] Ono LK, Qi YB, Liu SZ (2018). Progress toward stable lead halide perovskite solar cells. Joule.

[30] Kim YG, Shim CH, Kim DH, Lee HJ, Lee HJ (2012). Fabrication of transparent conductive oxide-less dye-sensitized solar cells consisting of Ti electrodes by electron-beam evaporation process. Thin Solid Films.

[31] Zhang C, Wang S, Zhang H, Feng Y, Tian W (2019). Efficient stable graphene-based perovskite solar cells with high flexibility in device assembling via modular architecture design. Energy Environ. Sci..

[32] Sui KR, Shi YW, Tang XL, Zhu XS, Iwai K (2008). Optical properties of AgI/Ag infrared hollow fiber in the visible wavelength region. Opt. Lett..

[33] Cave JM, Courtier NE, Blakborn IA, Jones TW (2020). Deducing transport properties of mobile vacancies from perovskite solar cell characteristics. J. Appl. Phys..

[34] Yanagida M, Shirai Y, Khadka DB, Miyano K (2020). Photoinduced ion-redistribution in CH_3_NH_3_PbI_3_ perovskite solar cells. Phys. Chem. Chem. Phys..

[35] Li C, Guerrero A, Huettner S, Bisquert J (2018). Unravelling the role of vacancies in lead halide perovskite through electrical switching of photoluminescence. Nat. Commun..

[36] Velilla E, Jaramillo F, Mora-Sero I (2021). High-throughput analysis of the ideality factor to evaluate the outdoor performance of perovskite solar minimodules. Nat. Energy.

[37] Nakane A, Tampo H, Tamakoshi M, Fujimoto S, Kim KM (2016). Quantitative determination of optical and recombination losses in thin-film photovoltaic devices based on external quantum efficiency analysis. J. Appl. Phys..

[38] Liu J, Bastiani MD, Aydin E, Harrison GT, Gao Y (2022). Efficient and stable perovskite-silicon tandem solar cells through contact displacement by MgF_X_. Science.

[39] J. Nelson, The physics of solar cells. (Imperial College Press, 2003). 10.1142/p276

[40] Back H, Kim G, Kim J, Kong J, Kim TK (2016). Achieving long-term stable perovskite solar cells via ion neutralization. Energy Environ. Sci..

[41] Wu SH, Chen R, Zhang SS, Babu BH, Yue YF (2019). A chemically inert bismuth interlayer enhances long-term stability of inverted perovskite solar cells. Nat. Commun..

[42] Kaushik VK (1991). XPS core level spectra and auger parameters for some silver compounds. J. Electron Spectros. Relat. Phenom..

[43] Tiller WA, Jackson KA, Rutter JW, Chalmers B (1953). The redistribution of solute atoms during the solidification of metals. Acta Metall..

[44] Yagi K, Minoda H, Degawa M (2001). Step bunching, step wandering and faceting: self-organization at Si surfaces. Surf. Sci. Rep..

[45] Zaluska-Kotur MA, Krzyzewski F (2012). Step bunching process induced by the flow of steps at the sublimated crystal surface. J. Appl. Phys..

[46] Morrod JK, Graham TCM, Prior KA, Cavenett BC (2005). N-type doping of zinc selenide using a silver iodide thermal effusion source. J. Crys. Growth.

[47] Chebbi M, Azambre B, Cantrel L, Koch A (2016). A combined drifts and DR-UV–Vis spectroscopic in situ study on the trapping of CH_3_I by silver-exchanged faujasite zeolite. J. Phys. Chem. C.

[48] Kyaw AKK, Wang DH, Wynands D, Zhang J, Thuc-Quyen N (2013). Improved light harvesting and improved efficiency by insertion of an optical spacer (ZnO) in solution-processed small-molecule solar cells. Nano Lett..

[49] Liao JY, Lei BX, Chen HY, Kuang DB, Su CY (2012). Oriented hierarchical single crystalline anatase TiO_2_ nanowire arrays on ti-foil substrate for efficient flexible dye-sensitized solar cells. Energy Environ. Sci..

