# Supplementary material for: An In-Situ Formed Tunneling Layer Enriches the Options of Anode for Efficient and Stable Regular Perovskite Solar Cells
Source: Nanomicro Lett. 2022 Dec 9;15:10. doi: 10.1007/s40820-022-00975-6 (PMC9733774; doi:10.1007/s40820-022-00975-6)
Supplement: Supplementary file 1 — Supplementary file1 (PDF 2238 KB) [file 40820_2022_975_MOESM1_ESM.pdf]

Supporting Information for

An In-Situ Formed Tunneling Layer Enriches the Options of Anode for Efficient and Stable Regular Perovskite Solar Cells

Xuesong Lin[1], Yanbo Wang[1,*], Hongzhen Su[1], Zhenzhen Qin[1], Ziyang Zhang[1], Mengjiong Chen[1], Min Yang[1], Yan Zhao[1], Xiao Liu[2], Xiangqian Shen[1], Liyuan Han[1,2,*]

[1]State Key Laboratory of Metal Matrix Composites, Shanghai Jiao Tong University, Shanghai 200240, P. R. China

[2]Special Division of Environmental and Energy Science, Komaba Organization for Educational Excellence, College of Arts and Sciences, University of Tokyo, Tokyo 153-8902, Japan

*Corresponding authors. E-mail: han.liyuan@sjtu.edu.cn (Liyuan Han); sjtu-wyb@sjtu.edu.cn

(Yanbo Wang)

Note S1: Key Parameters Affecting PCE in Regular Ag-PSCs

The influence of each parameter (P) by dark storage on the PCE of devices be evaluated by an improvement index (ΔI), as shown in Eq. S1. After 48h stored under dark and dry environment, the PCE showed a ΔI of 25.3%, with ΔIs of 1.4%, 0.5% and 22.8% for V_{OC}, J_{SC} and FF, respectively. Moreover, the FF as the key parameters affecting PCE was enhanced mainly by the halved R_s (Fig. S2E), which normally related to the improvement of interface charge transport and collection. As shown in Fig. S1, the first 24 h for storage was critical in which period the major improvement of PCE happened.

$$\Delta I = \frac{P(48h-f)+P(48h-r)-P(0h-f)+P(0h-r)}{P(0h-f)+P(0h-r)} \quad (S1)$$

In detail, when we consider the solar cell system as an equivalent circuit with parasitic resistance (Fig. S2A), the parasitic resistance includes R_s and R_{sh}, and the volt-ampere characteristics can be described by Eq. S2.

$$J = J_{SC} - J_0\{\exp\left[q\left(V + \frac{AJ(V)R_s}{k_B T_a}\right)\right] - 1\} - \frac{V+AJ(V)R_s}{AR_{sh}} \quad (S2)$$

Where J_0, T_a and A are the dark current density, atmosphere temperature and active area, respectively. The q and k_B are the elemental charge and Boltzmann constant, respectively. When the reduction of leakage current mainly originates from the reduction of R_s, the simulated J-V curve shows a large enhancement of FF without significant changes in J_{SC} and V_{OC}, as shown in Fig. S2B. The simulated results are well matched with our experimental results in **Fig. 1A-C** and Figure S1.

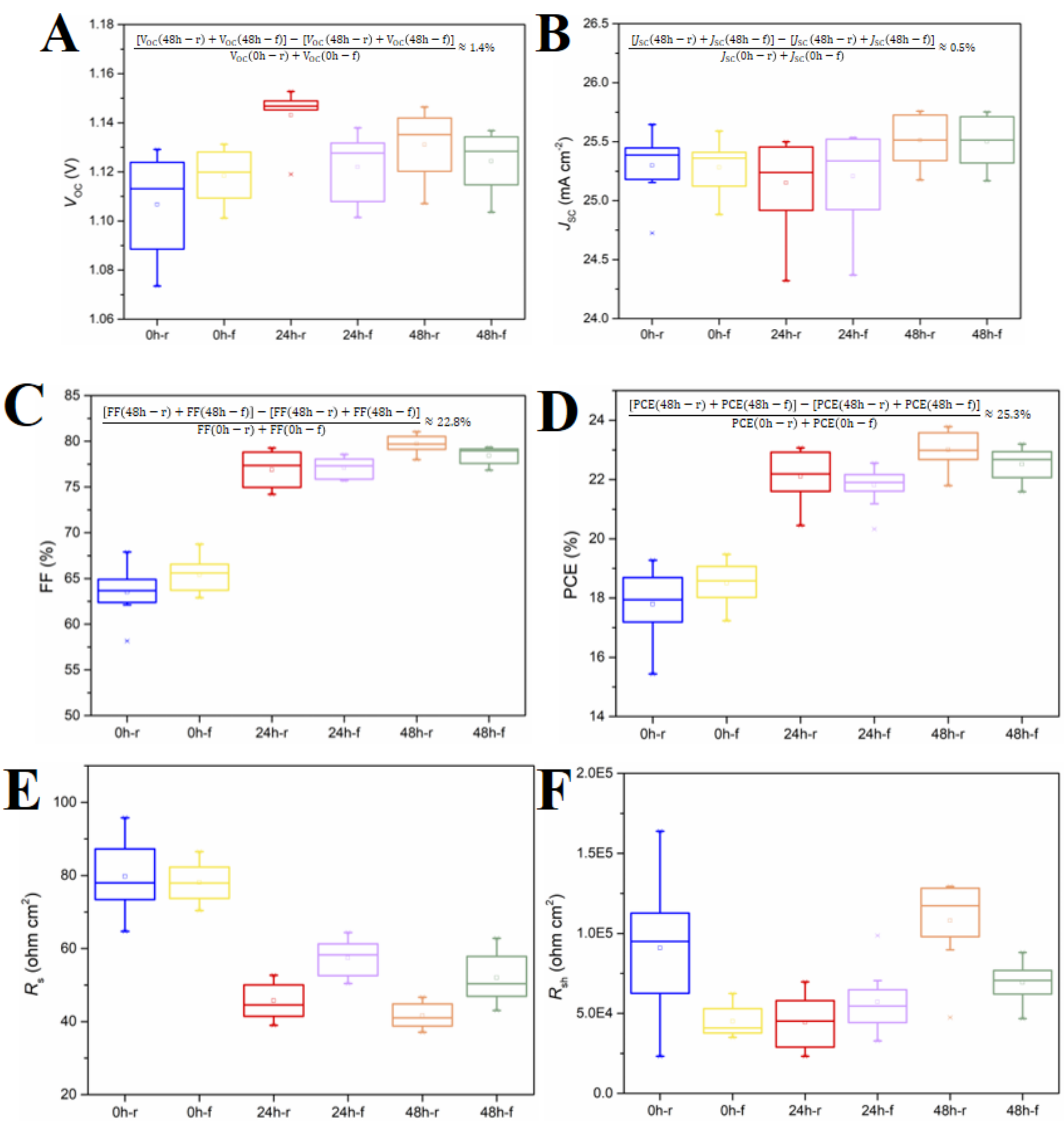

Fig. S1 Original performance evolution of regular Ag-PSCs stored under dark and dry environment. (**A**) V_{OC}, (**B**) J_{SC}, (**C**) FF, (**D**) PCE, (**E**) R_s and (**F**) R_{sh}. Twenty individual PSCs were fabricated, and the -f and -r represent the J-V characterization under forward and reverse scan, respectively

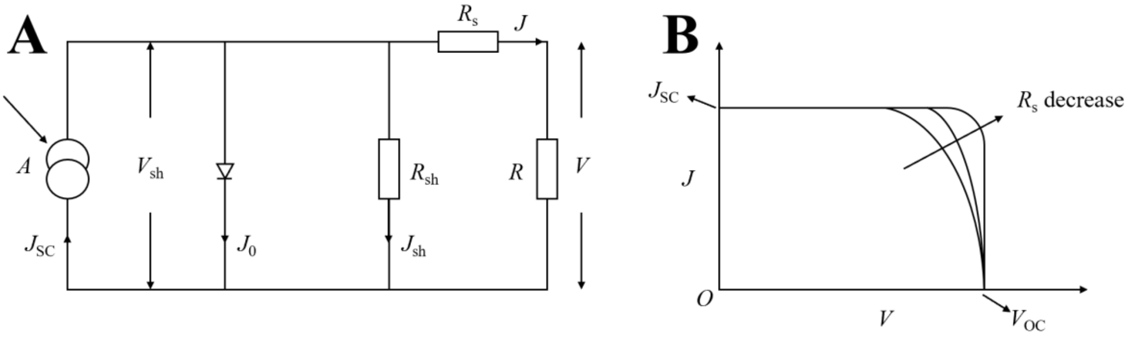

Fig. S2 The Equivalent circuit of a solar cell with parasitic resistance. (**A**) schematic diagram and (**B**) the effect of reduction of R_s on the J-V curves. The V_{sh} and J_{sh} is the shunt voltage and shunt current density, respectively

Note S2: The Reason for Selecting (FA,MA)Pb(I,Br)$_3$ Perovskite System

The similar performance enhancement in FFs was also observed in regular Ag-devices based on (FA,MA)Pb(I,Br)$_3$, and the critical period for enhancement was also the first 24 h of storage. These phenomena suggest the following points: 1) the efficient regular Ag-PSCs can be realized with various record-maker perovskites; 2) the main reason for the enhancement of FF and PCE should be the self-modification of the adjacent interface of HTM/Ag anode in the first 24 h.

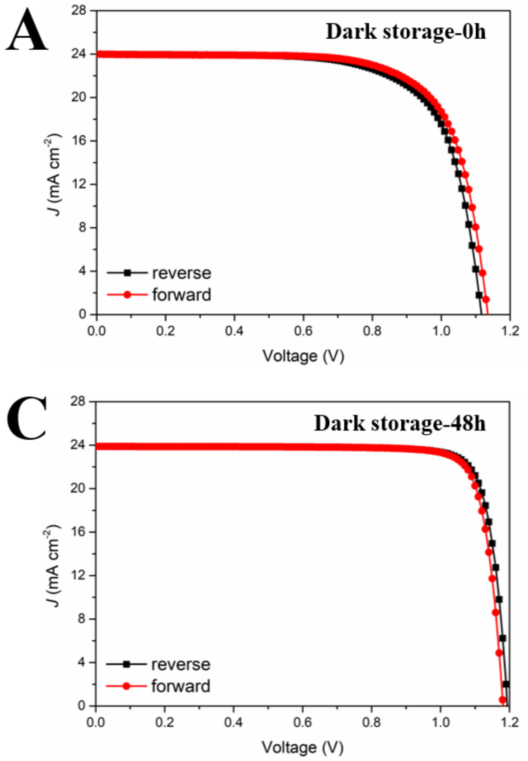

Fig. S3 *J*-*V* curves of champion regular Ag-PSCs based on (FA,MA)Pb(I,Br)$_3$ stored under dark and dry environment for varying times. (**A**) 0h, (**B**) 24h, and (**C**) 48h

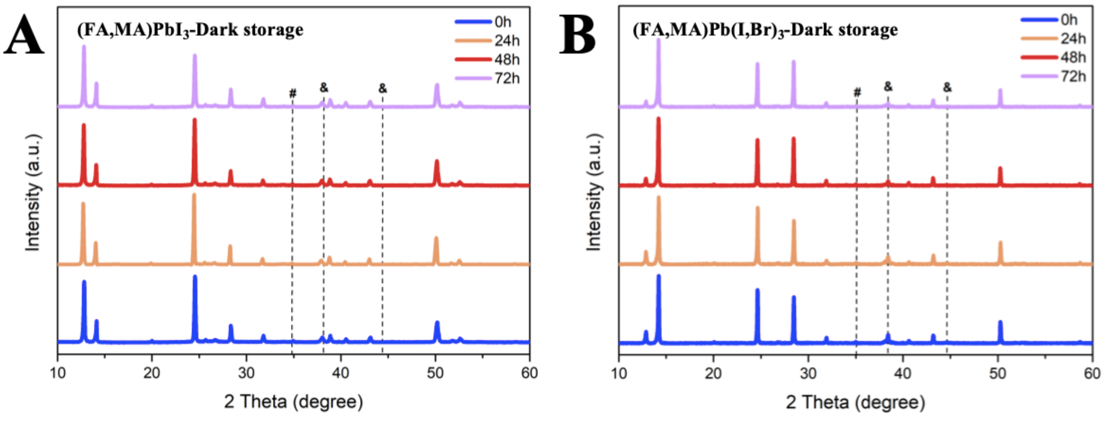

Fig. S4 XRD patterns of perovskite on regular Ag-PSCs stored under dark and dry environment for varying times. (**A**) (FA,MA)PbI$_3$ perovskite and (**B**) (FA,MA)Pb(I,Br)$_3$ perovskite. Notes that the Ag anode was peeled off by tape. The characteristic peaks of residue AgI and Ag are marked with signature # and &, respectively

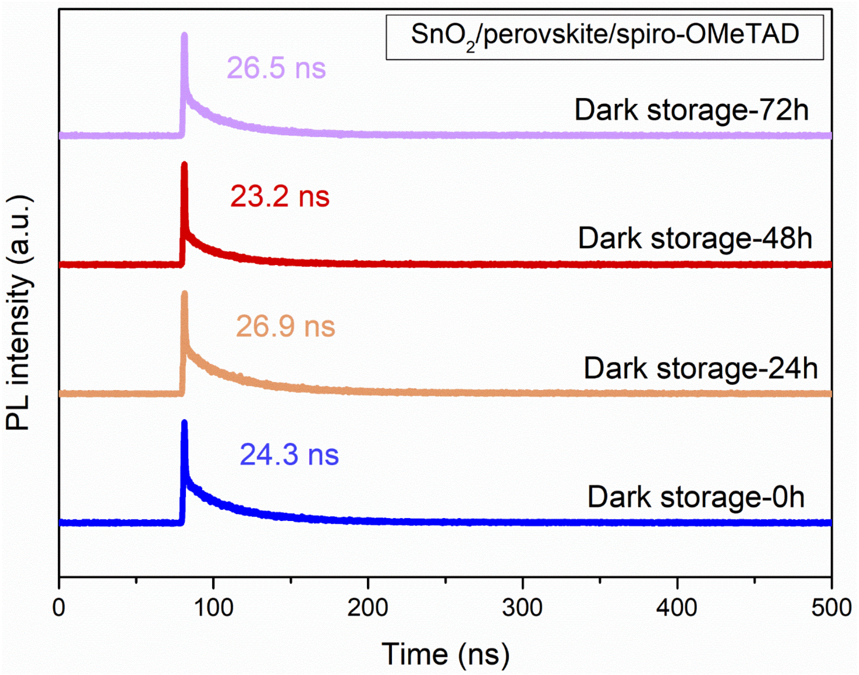

Fig. S5 TRPL results of SnO_2/(FA,MA)PbI_3 perovskite/spiro-OMeTAD bi-interfaces on glass substrates stored under dark and dry environment for varying times

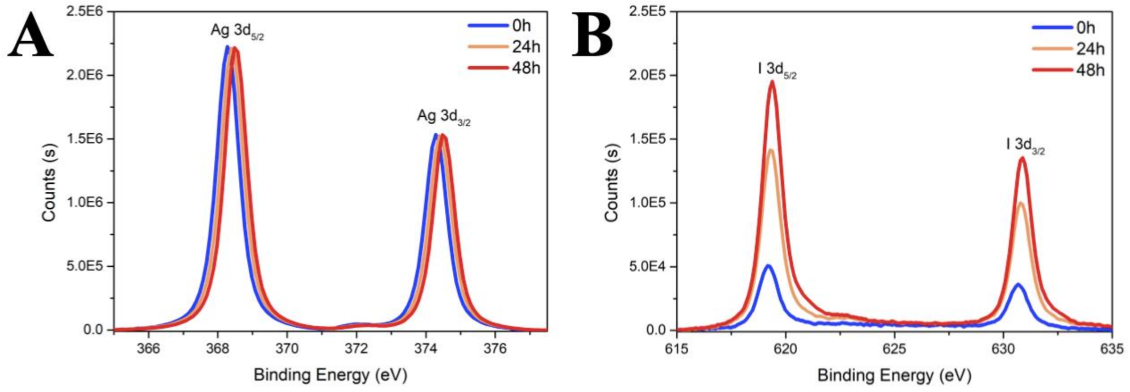

Fig. S6 High-resolution XPS of Ag anode on PSCs stored under dark and dry environment (**A**) Ag 3d and (**B**) I 3d core signals

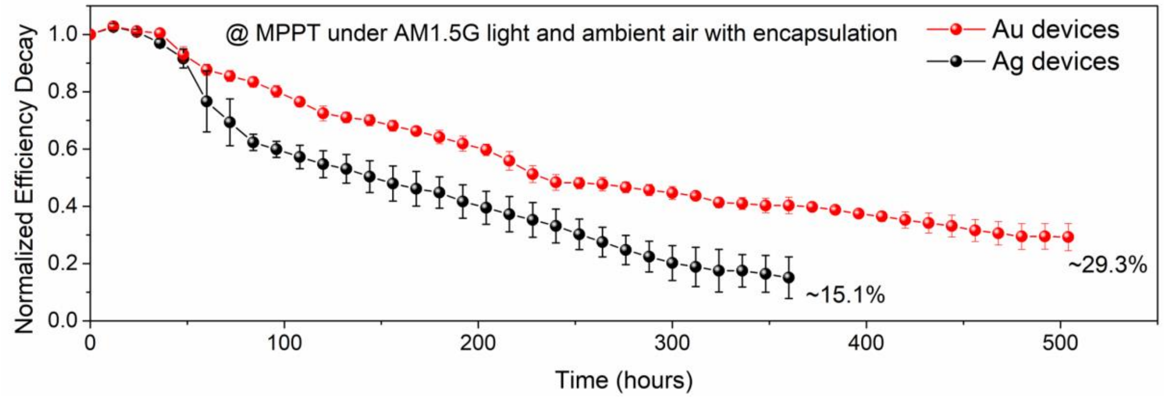

Fig. S7 The operational stability of encapsulated Ag and Au devices at MPP under 1-sun illumination (AM 1.5G, 100 mW cm^{-2}) and ambient air. The error bars denote standard deviations for five individual devices

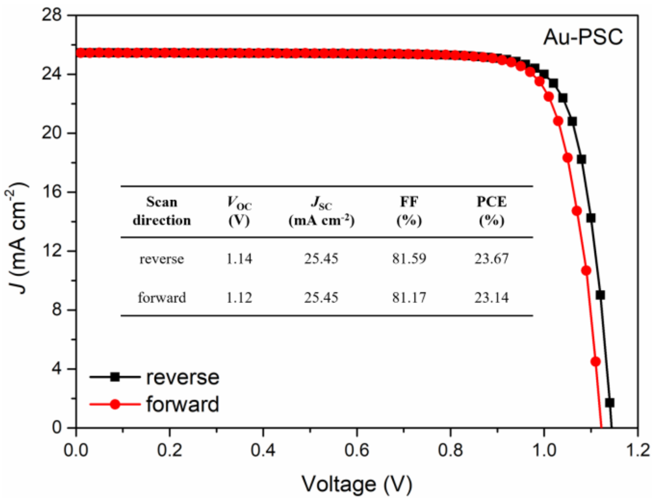

Scan direction	V_{OC} (V)	J_{SC} (mA cm^{-2})	FF (%)	PCE (%)
reverse	1.14	25.45	81.59	23.67
forward	1.12	25.45	81.17	23.14

Fig. S8 *J*-*V* curves of champion regular Au-PSC

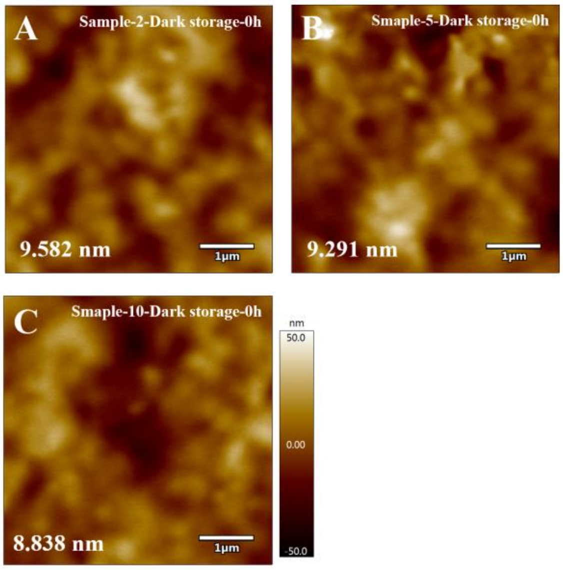

Fig. S9 The xy-plane film morphology of glass/FTO/SnO_2/(FA,MA)PbI_3 perovskite/spiro-OMeTAD/ultrathin Ag layer without storage by depositing (**A**) 2nm, (**B**) 5nm, and (**C**) 10nm, respectively, measured by AFM

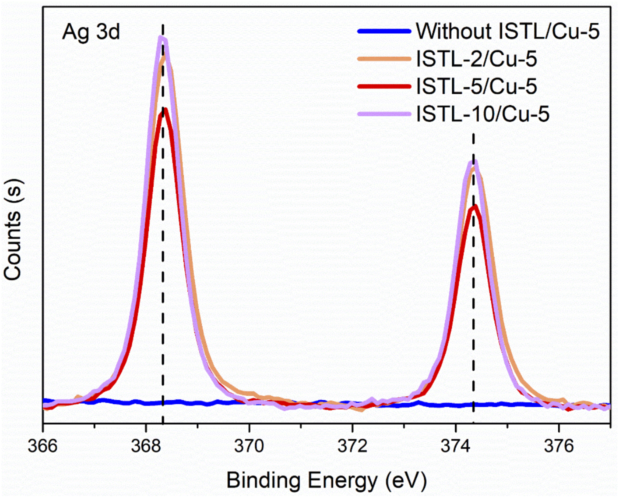

Fig. S10 High-resolution XPS of Ag 3d core levels of the evaporated Ag atop FTO/SnO_2/(FA,MA)PbI_3 perovskite/spiro-OMeTAD after 24-hour storage under dark and dry environment (< 10% relative humidity)

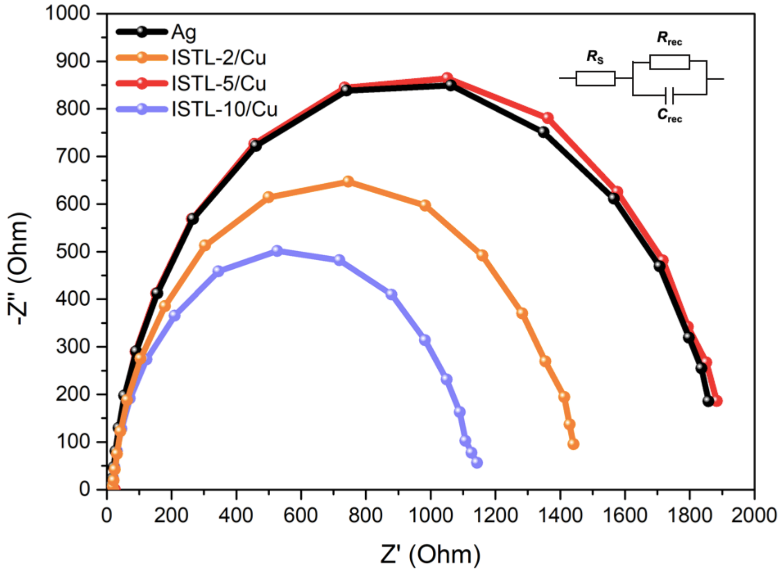

Fig. 11 Nyquist plots of Ag-PSC and Cu-PSCs with different ISTLs in dark condition with 0.9 V bias around 1 Hz to 1 MHz. The equivalent circuit employed to fit the spectra, where R_S is the series resistance and C_{rec} is charge recombination capacitance

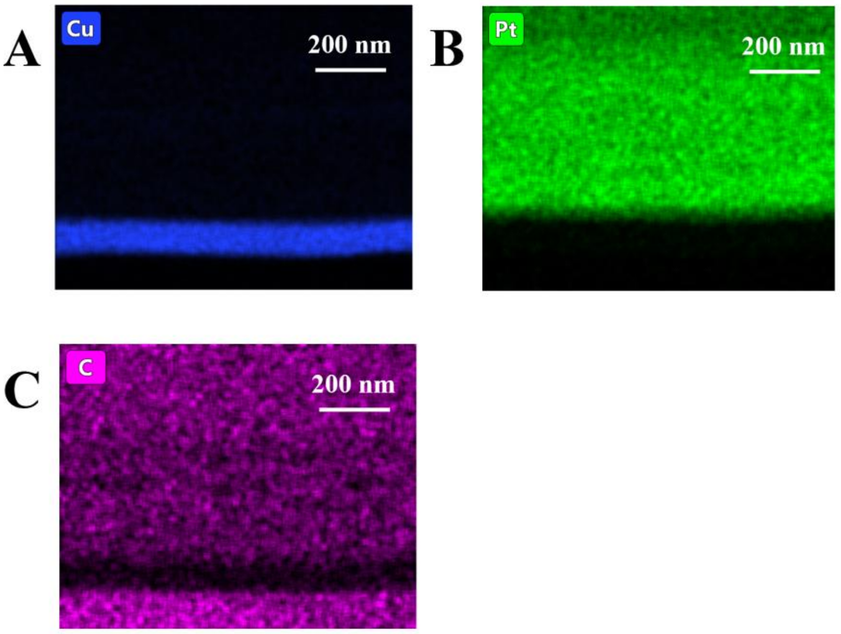

Fig. S12 Element distributions of ISTL-5 in Figure 4B by EDS mapping. (**A**) Cu element, (**B**) Pt element and (**C**) C element

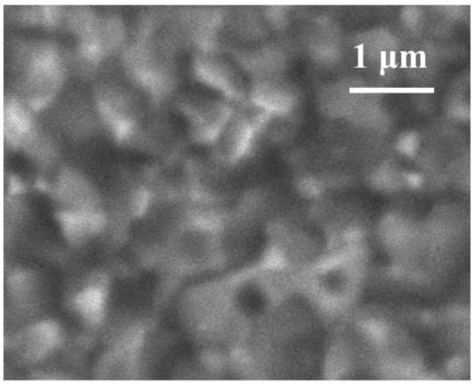

Fig. S13 The top-view SEM image of ISTL-5 atop FTO/SnO_2/(FA,MA)PbI_3 perovskite/spiro-OMeTAD

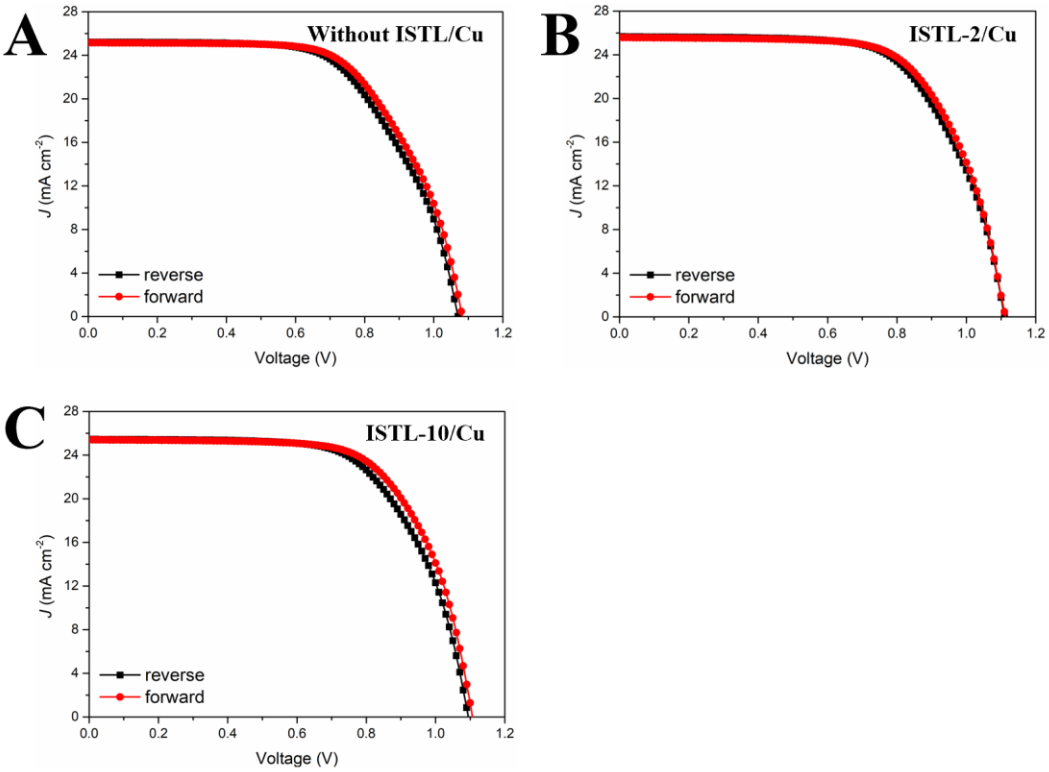

Fig. S14 J-V curves of champion Cu-PSCs (**A**) without ISTL, (**B**) with ISTL-2, and (**C**) with ISTL-10 stored under dark and dry environment for 48h, respectively

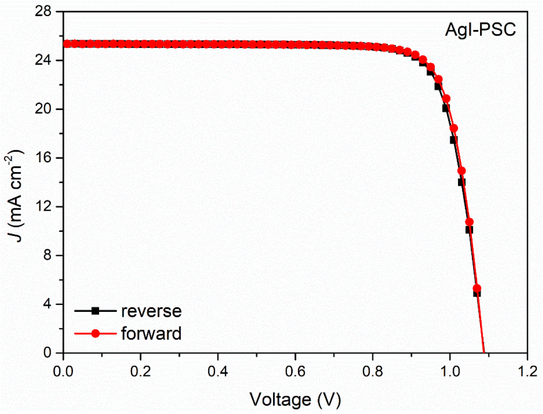

Fig. S15 *J*-*V* curves of the champion regular AgI-PSC

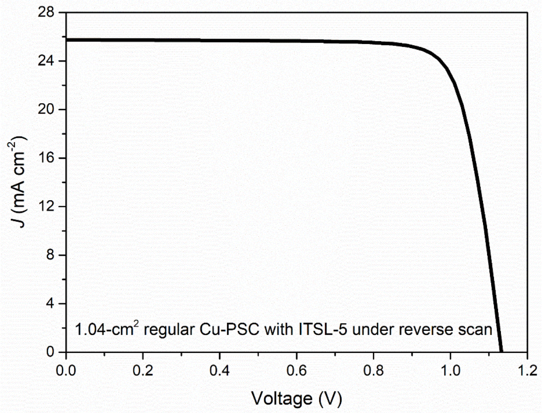

Fig. S16 *J*-*V* curves of the champion Cu-PSC with ISTL-5 and an aperture area of 1.04 cm^2 under reverse scan measured in our lab

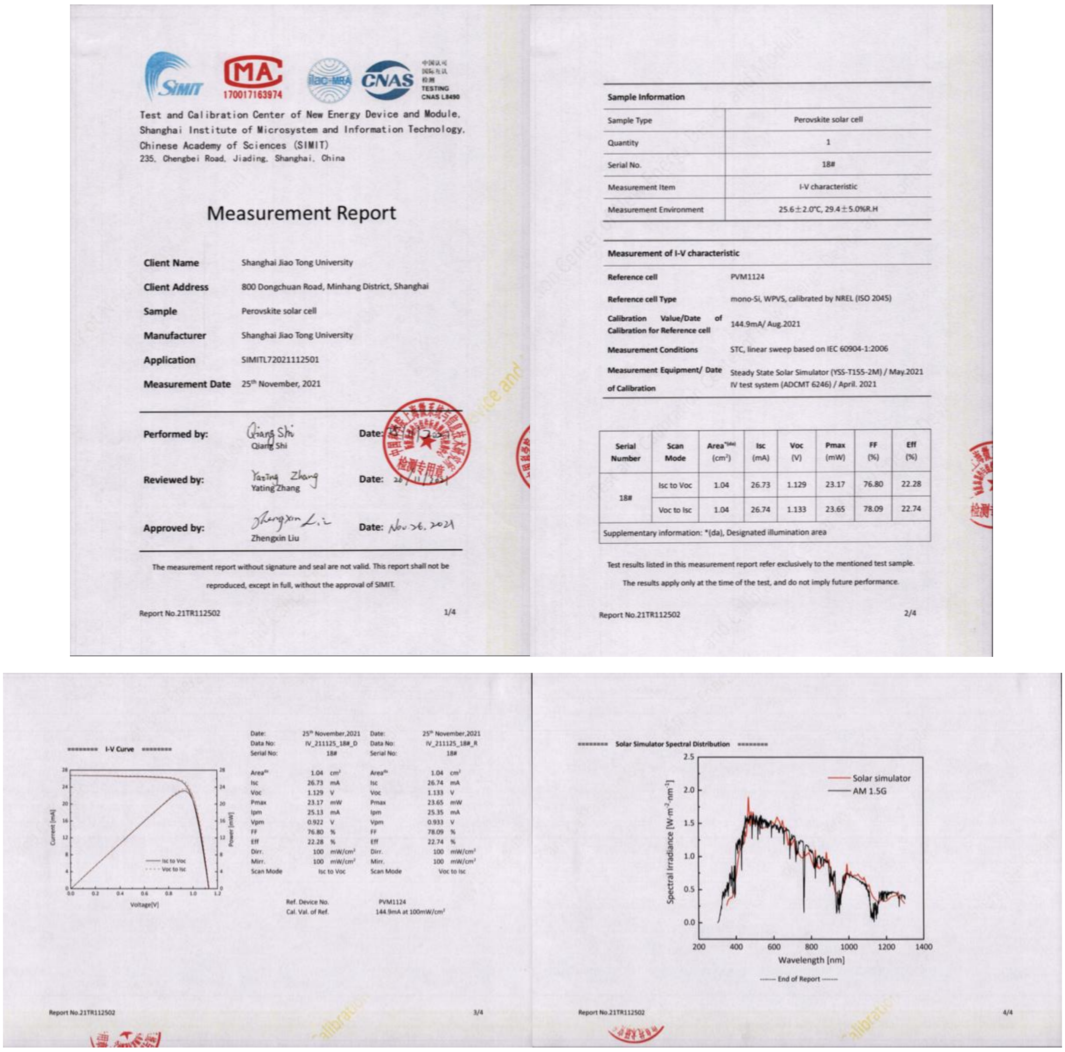

Test and Calibration Center of New Energy Device and Module, Shanghai Institute of Microsystem and Information Technology, Chinese Academy of Sciences (SIMIT)
235, Chengbei Road, Jiading, Shanghai, China

Measurement Report

Client Name	Shanghai Jiao Tong University
Client Address	800 Dongchuan Road, Minhang District, Shanghai
Sample	Perovskite solar cell
Manufacturer	Shanghai Jiao Tong University
Application	SIMITL72021112501
Measurement Date	25th November, 2021

Performed by: Qiang Shi Date: [illegible]
Reviewed by: Yating Zhang Date: 26/11/2021
Approved by: Zhengxin Liu Date: Nov. 26, 2021

The measurement report without signature and seal are not valid. This report shall not be reproduced, except in full, without the approval of SIMIT.

Report No.21TR112502 1/4

Sample Information	
Sample Type	Perovskite solar cell
Quantity	1
Serial No.	18#
Measurement Item	I-V characteristic
Measurement Environment	25.6±2.0℃, 29.4±5.0%R.H

Measurement of I-V characteristic	
Reference cell	PVM1124
Reference cell Type	mono-Si, WPVS, calibrated by NREL (ISO 2045)
Calibration Value/Date of Calibration for Reference cell	144.9mA/ Aug.2021
Measurement Conditions	STC, linear sweep based on IEC 60904-1:2006
Measurement Equipment/ Date of Calibration	Steady State Solar Simulator (YSS-T155-2M) / May.2021<br>IV test system (ADCMT 6246) / April. 2021

Serial Number	Scan Mode	Area*(da) (cm²)	Isc (mA)	Voc (V)	Pmax (mW)	FF (%)	Eff (%)
18#	Isc to Voc	1.04	26.73	1.129	23.17	76.80	22.28
	Voc to Isc	1.04	26.74	1.133	23.65	78.09	22.74
Supplementary information: *(da), Designated illumination area							

Test results listed in this measurement report refer exclusively to the mentioned test sample.
The results apply only at the time of the test, and do not imply future performance.

Report No.21TR112502 2/4

======== I-V Curve ========

Current [mA]; Power [mW]; Voltage[V]; Isc to Voc; Voc to Isc

Date:	25th November,2021	Date:	25th November,2021
Data No:	IV_211125_18#_D	Data No:	IV_211125_18#_R
Serial No:	18#	Serial No:	18#
Area	1.04 cm²	Area	1.04 cm²
Isc	26.73 mA	Isc	26.74 mA
Voc	1.129 V	Voc	1.133 V
Pmax	23.17 mW	Pmax	23.65 mW
Ipm	25.13 mA	Ipm	25.35 mA
Vpm	0.922 V	Vpm	0.933 V
FF	76.80 %	FF	78.09 %
Eff	22.28 %	Eff	22.74 %
Dirr.	100 mW/cm²	Dirr.	100 mW/cm²
Mirr.	100 mW/cm²	Mirr.	100 mW/cm²
Scan Mode	Isc to Voc	Scan Mode	Voc to Isc

Ref. Device No. PVM1124
Cal. Val. of Ref. 144.9mA at 100mW/cm²

Report No.21TR112502 3/4

======== Solar Simulator Spectral Distribution ========

Solar simulator; AM 1.5G; Spectral irradiance [W·m⁻²·nm⁻¹]; Wavelength [nm]

---- End of Report ----

Report No.21TR112502 4/4

Fig. S17 Certificated results of 1.04-cm² Cu-PSC with ISTL-5 by the Shanghai Institute of Microsystem and Information Technology, Chinese Academy of Science, China (SIMIT, China). The reverse scan is performed from 1.2 V to –0.1 V at 30 mV s^{-1}, with a PCE of 22.74% (V_{OC}=1.133 V, I_{SC}=26.74 mA, FF=78.09%). The forward scan is performed from –0.1 V to 1.2 V at 30 mV s^{-1}, with a PCE of 22.28% (V_{OC}=1.129 V, I_{SC}=26.73 mA, FF=76.80%). The hysteresis index of the device is less than 3%

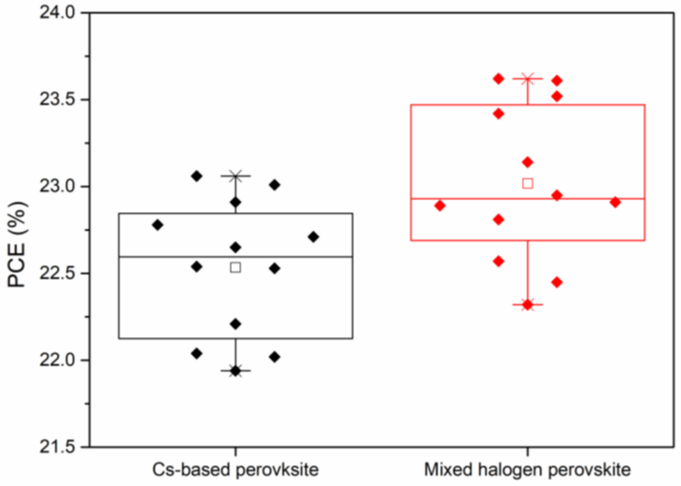

Fig. S18 The box plots of PCE of 0.09-cm² Cu-PSCs with different perovskites stored under dark and dry environment for 48h. The device structure is FTO/SnO_2/perovskite/spiro-OMeTAD/ISTL-5/Cu

Table S1 Performance parameters of champion regular Ag-PSC stored under dark and dry environment for varying times

Dark Storage Time (h)	V_{OC} (V)	J_{SC} (mA cm^{-2})	FF (%)	PCE (%)	R_s (Ohm cm^2)	R_{sh} (Ohm cm^2)
0-r	1.11	25.37	67.91	19.12	5.18	5617.11
0-f	1.12	25.32	68.76	19.50	5.86	2804.26
24-r	1.15	25.50	79.01	23.17	3.41	5571.82
24-f	1.12	25.52	77.70	22.21	5.06	4187.49
48-r	1.14	25.71	81.04	23.86	2.97	10344.49
48-f	1.12	25.69	79.50	22.87	4.88	5344.84

Table S2 Performance parameters of champion Ag-PSC based on (FA,MA)Pb(I,Br)$_3$ stored under dark and dry environment for varying times

Dark Storage Time (h)	V_{OC} (V)	J_{SC} (mA cm^{-2})	FF (%)	PCE (%)	R_s (Ohm cm^2)	R_{sh} (Ohm cm^2)
0-r	1.12	24.00	69.91	18.79	3.92	4758.80
0-f	1.13	23.98	70.81	19.19	3.77	3284.07
24-r	1.17	23.88	83.32	23.28	2.15	16787.16
24-f	1.17	23.85	81.84	22.84	2.84	26826.16
48-r	1.19	23.87	83.33	23.67	1.90	18440.14
48-f	1.18	23.87	83.44	23.51	2.17	18097.96

Table S3 The estimated t_{ave} of iodine ions through spiro-OMeTAD HTMs with a thickness ranging from 100 nm to 300 nm

The thickness of spiro-OMeTAD HTM (nm)	Estimated t_{ave} (hour)
100	0.031
150	0.070
200	0.124
250	0.195
300	0.280

Table S4 Performance parameters of champion Cu-PSCs without ISTL or with different thickness of ISTLs stored under dark and dry environment for 48h

Sample	V_{OC} (V)	J_{SC} (mA cm^{-2})	FF (%)	PCE (%)	R_s (Ohm cm^2)	R_{sh} (Ohm cm^2)
Without ISTL/Cu-r	1.07	25.21	62.12	16.76	6.63	13106.25
Without ISTL/Cu-f	1.08	25.15	63.45	17.23	6.50	4997.98
ISTL-2/Cu-r	1.11	25.66	65.41	18.63	5.83	7053.58
ISTL-2/Cu-f	1.11	25.58	66.71	18.94	5.93	3036.72
ISTL-5/Cu-r	1.11	25.68	82.95	23.64	2.30	32120.48
ISTL-5/Cu-f	1.11	25.69	82.36	23.51	2.20	20572.67
ISTL-10/Cu-r	1.09	25.45	64.40	17.86	5.91	8150.05
ISTL-10/Cu-f	1.11	25.41	66.42	18.73	5.63	3388.79

Table S5 Performance parameters of champion Cu-PSC with ISTL-5 stored under dark and dry environment for varying times

Dark Storage Time (h)	V_{OC} (V)	J_{SC} (mA cm^{-2})	FF (%)	PCE (%)	R_s (Ohm cm^2)	R_{sh} (Ohm cm^2)
0-r	1.09	25.72	81.58	22.87	2.90	35794.66
0-f	1.08	25.69	80.59	22.36	2.01	12564.78
24-r	1.11	25.69	82.16	23.43	2.30	17414.20
24-f	1.10	25.70	81.16	22.94	2.12	10683.22
48-r	1.11	25.68	82.95	23.64	2.30	32120.48
48-f	1.11	25.69	82.36	23.51	2.20	20572.67

Table S6 Performance parameters of champion regular PSCs with other ISTLs or low-WF anodes stored under dark and dry environment for 48h

Sample	V_{OC} (V)	J_{SC} (mA cm^{-2})	FF (%)	PCE (%)
ISTL-CdI_2/Cu-r	1.08	25.07	81.63	22.10
ISTL-CdI_2/Cu-f	1.08	25.06	80.25	21.72
ISTL-SnI_4/Cu-r	1.10	25.06	80.05	22.07
ISTL-SnI_4/Cu-f	1.09	25.07	78.73	21.51
ISTL-AgI/Ti-r	1.10	25.02	80.04	22.03
ISTL-AgI/Ti-f	1.09	25.01	78.25	21.33
ISTL-AgI/Al-r	1.09	25.22	80.21	22.05
ISTL-AgI/Al-f	1.07	25.24	78.43	21.18
